# Designing stepped wedge trials to evaluate physical activity interventions in schools: methodological considerations

**DOI:** 10.1186/s12966-025-01720-z

**Published:** 2025-02-25

**Authors:** Ruth Salway, Danielle House, Simona Kent-Saisch, Robert Walker, Lydia Emm-Collison, Alice Porter, David R Lubans, Michael Beets, Frank de Vocht, Russell Jago

**Affiliations:** 1 Population Health Sciences, Bristol Medical School, University of Bristol, Bristol, UK; 2 Centre for Exercise, Nutrition and Health Sciences, University of Bristol, Bristol, UK; 3 NIHR Bristol Biomedical Research Centre, University Hospitals Bristol and Weston NHS Foundation Trust and University of Bristol, Bristol, UK; 4 Centre for Active Living and Learning, College of Human and Social Futures, University of Newcastle, Callaghan, NSW Australia; 5 Hunter Medical Research Institute, New Lambton Heights, NSW 2305 Australia; 6 Faculty of Sport and Health Sciences, University of Jyväskylä, Jyväskylä, Finland; 7 Arnold School of Public Health, University of South Carolina, Columbia, SC USA; 8 Applied Research Collaboration West (NIHR ARC West), The National Institute for Health Research, University Hospitals Bristol and Weston NHS Foundation Trust, Bristol, UK

## Abstract

**Background:**

In a stepped wedge design, schools are randomised to a sequence of measurements, with each sequence transitioning to intervention status at a different time. There are several advantages to such designs, including increased statistical power, logistical benefits and the ability to explore change over time. However, stepped wedge designs have not previously been used to evaluate school-based physical activity interventions in children. This paper aimed to explore the feasibility of this design, by identifying school constraints, balancing these with statistical considerations and exploring the power of this chosen design under different scenarios.

**Methods:**

We conducted three interlinked studies, with the results from one informing the next. Study 1 was a qualitative study to identify school constraints that inform the choice of stepped wedge configuration. Study 2 used simulation to choose a configuration that balanced these school constraints and statistical properties. Study 3 explored the statistical power for the chosen design for different school and pupil sample sizes, using an open cohort design (a mixture of new and repeated pupils).

**Results:**

School staff considered the proposed data collection feasible, and supported a maximum of 3–4 measurements per year and an implementation period of one school term. Study 2 therefore considered incomplete stepped wedge designs with five steps. Statistically, the best designs had a mix of control and intervention measurements in terms 2–4 and a spread of measurements across the whole study duration. Power depended on a combination of the overall recruitment rate and the retention rate. For 20 schools with an eligible class size of 30 pupils, we would be able to detect a 6 min difference in average weekday moderate-to-vigorous physical activity with 80% power, provided there were > 50% of pupils measured per school at each time. A similarly powered cluster randomised controlled trial would require 42 schools.

**Conclusion:**

Stepped wedge trials are a viable design for evaluating school-based physical activity interventions. Incomplete designs, where not all schools are measured at each point, offer the flexibility to work around practical constraints.

**Supplementary Information:**

The online version contains supplementary material available at 10.1186/s12966-025-01720-z.

## Introduction

Increasing children’s physical activity is important for their mental wellbeing and physical health [1]. While schools are ideal settings to promote and provide opportunities for physical activity, to date the impact of school-based interventions on physical activity has been limited [2, 3]. In a previous paper [4] we identified two key issues with current approaches. Firstly, most interventions do not take into account the school context such as existing programmes, facilities, logistical challenges, ethos, and demographics, which can contribute to poor implementation. Secondly, only a limited range of evaluation designs are currently used. The latter is the focus of this paper. Traditionally, school-based interventions are typically evaluated using a clustered randomised controlled trial design (CRCT), which focuses on internal validity and provides a robust estimate of the intervention effect under the exchangeability assumption [5]. However, for school-based research, practical issues such as lack of blinding, differential loss to follow-up, and imbalance between control and intervention groups can affect both the accuracy and precision of estimates [5, 6]. Whole school approaches that consider school context have been proposed [7, 8] but evaluation of such complex interventions need to address a broader range of questions than CRCT designs allow, and MRC guidance advocates for a wider range of designs [9]. This also aligns with the practice of triangulation from aetiological epidemiology [10], where results are integrated across different designs, each with different strengths and sources of potential bias. Complex interventions are a specific example where the strategic combination of multiple approaches may produce more robust, nuanced and generalisable results.

**Fig. 1:**
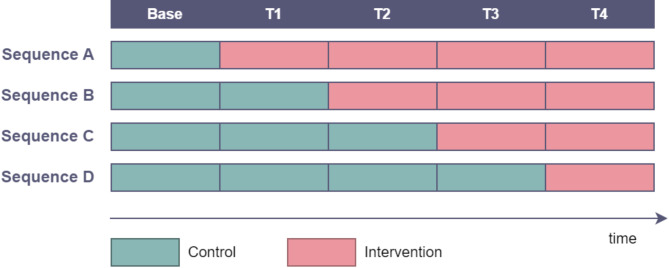
Generic example of a stepped wedge trial

Stepped wedge designs [11, 12] are a type of cluster randomised trial, but instead of randomising to intervention or control arms, clusters are randomised to a sequence of control and intervention states over time (Fig. 1). All clusters start under control conditions, and then each sequence transitions to intervention status at a different point in time, with all clusters eventually receiving the intervention. The strength of this design is in repeated measures, as each cluster is measured at multiple times under different conditions. Individuals within clusters may be different at each time point (cross-sectional), the same (closed cohort) or a mixture of new and repeated individuals (open cohort). Recent work [13, 14] has explored incomplete stepped wedge designs where measurements are not taken for all sequences at all time points, for example to allow for an intervention implementation period.

There are a number of advantages to the stepped wedge design. In general, fewer clusters are needed than for a similarly-powered CRCT, especially for cohort designs [15]. They can be logistically easier as intervention implementation support is spread across the study duration, and will often improve uptake as all clusters will eventually receive the intervention. From a statistical perspective, the repeated measures design allows exploration of within and between-cluster variability, between-cluster heterogeneity in intervention effects and the ability to explore how the intervention effect changes over time [16, 17]. It also provides more intervention measurements (whilst not increasing the total number of measurements), which can be useful for process evaluation and secondary analysis of intervention implementation. However, this design does require multiple measurements within the same school, which can place increased burden on schools and pupils, may lead to higher drop-out rates, and the assessment process itself can affect behaviour. From a statistical perspective, the design is partially confounded by time because early time points are more likely to be under control conditions while later time points are predominantly intervention measurements, and so care must be taken to account properly for time [18]. This makes both the design and analysis of stepped wedge trials more complex.

**Fig. 2:**
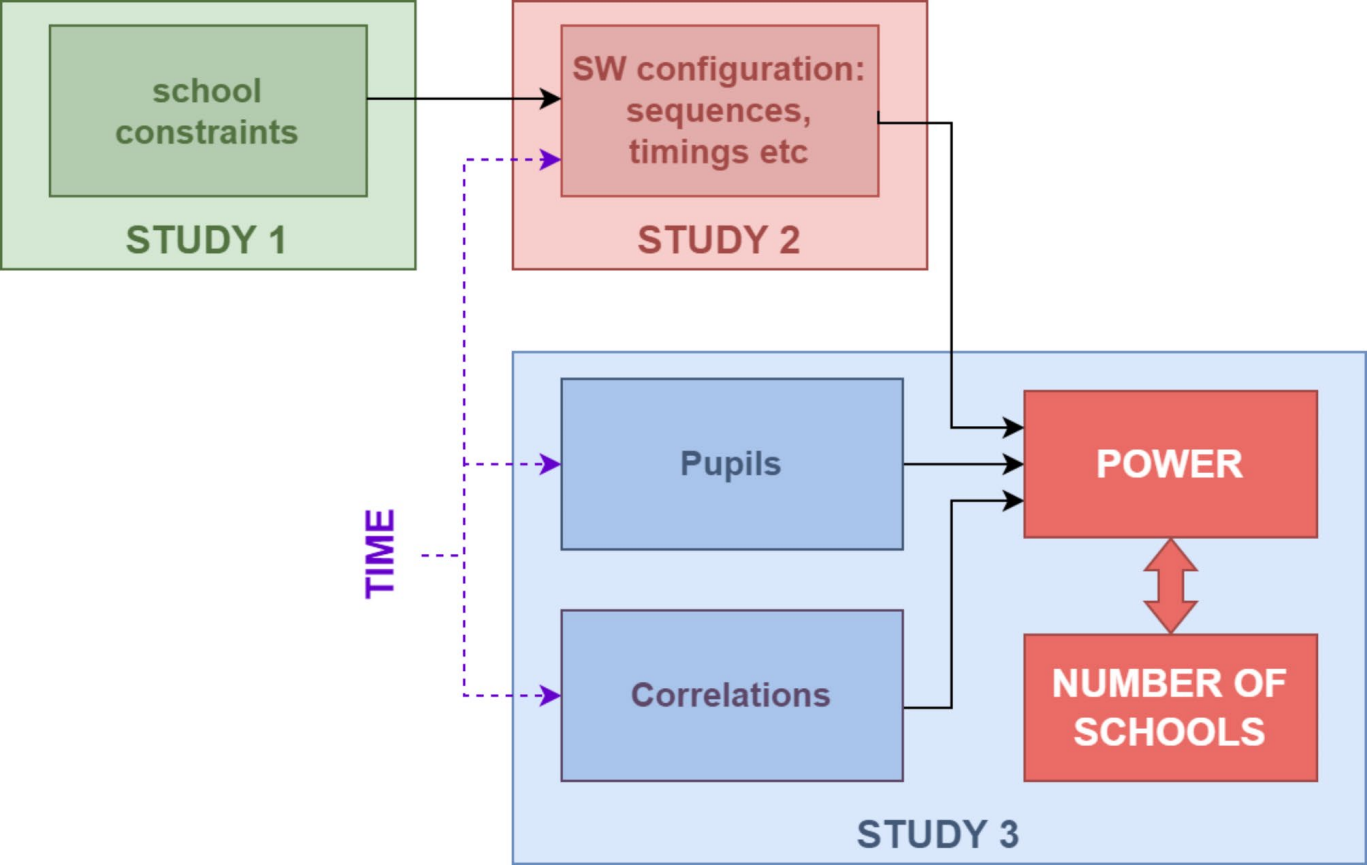
Elements that inform the power for a stepped wedge trial

To our knowledge stepped wedge designs have not yet been used to evaluate school-based physical activity interventions in children, possibly due to this increased complexity. In a CRCT, power depends on the number of pupils per school and the amount of clustering, via the intracluster correlation coefficient (ICC) [19]. Stepped wedge trials are more complex firstly because both of these may change over time and secondly because other factors affect power (Fig. 2). So, as well as the ICC, the power for a stepped wedge design depends on the number of pupils per school *at each time point*, the cluster autocorrelation over time (CAC), plus the specific stepped wedge design; that is, the number of sequences, allocation of schools to sequences, number of steps/measurement periods, and the timings of these [15]. For cohort designs, the power will additionally depend on the individual autocorrelation over time (IAC) and for an open cohort, it will also depend on the pattern of repeated versus new pupils over time [20, 21]. Approximate sample size formulae have been derived for many of these elements in isolation [15, 17, 22, 23, 24, 25], but calculations become more complex when multiple elements occur together. Moreover, much of the existing literature focuses on community or primary care settings, and it is not clear how school-specific constraints, such as school term structures or smaller eligible populations due to class sizes, may impact on the design. In addition, pupil physical activity exhibits strong seasonality in many countries, for example, with levels higher in summer than winter in the UK [26], and accounting for change over time is particularly important within a design where time is itself a confounder. These elements are all interlinked (Fig. 2). Power will thus depend on the specific configuration of sequences, steps, timings and measurements, which in turn is constrained by school preferences, logistical constraints and specific features of the intervention and/or outcome measures.

The aims of this paper are:

1. To identify school constraints that affect the choice of stepped wedge design for a school-based physical activity study;
2. To choose an appropriate stepped wedge design that reflects these and other constraints;
3. To calculate power for this chosen design under a range of scenarios and compare to the power of a similar-sized CRCT.

This paper consists of three interlinked studies as shown in Fig. 2, which correspond to the three aims. We present the three studies separately as the results from one inform the next. Study 1 is a qualitative study to explore school preferences and constraints that affect the stepped wedge design. Study 2 is a simulation study to compare different stepped wedge design configurations (that is, the specific configuration of sequences, steps, allocations, timings and measurement schedule) and select an appropriate configuration. Finally, Study 3 explores the statistical power for the chosen configuration via simulation. Table 1 provides a glossary of terms used throughout this paper.

**Table 1:** Glossary of terms

Term	Description
Intracluster correlation coefficient (ICC)	A measure of the amount of clustering within schools
Individual autocorrelation (IAC)	Correlation for the same pupil between two different time points
Cluster autocorrelation (CAC)	Correlation for the same school between two different time points
Cross-sectional design	Different pupils are measured at each time point
Closed cohort design	The same pupils are measured repeatedly over time points; pupils cannot join the study once it has started
Open cohort design	Pupils are repeated are measured repeatedly over time points, but pupils may join or leave the study at any time
Seasonal trend	Seasonal changes in outcome, the same from year to year
Secular trend	Changes in outcome over time
Sequence	A sequence of control and intervention statuses at each time period and whether measurement at this time occurs or not. Schools are randomly allocated to a sequence and sequences may have multiple schools allocated
Step	A planned time point when one or more schools switch from control to intervention status
Measurement period	The period of time covered by one step
Implementation period	A period of time to implement and fully embed the intervention
Stepped wedge configuration	the configuration of sequences, steps, allocations, timings and measurement schedule that form a specific stepped wedge design
Incomplete design	A stepped wedge design where measurements are only taken in some schools at each measurement point, rather than for all schools at all times.
Data generating model	Statistical model used to generate data in the simulation study
Analysis model	Statistical model used to analyse data in the simulation study

### Study context

This paper focuses specifically on primary schools (ages 4–11) in England, although the steps described can be replicated in other settings. In England, each year group in a state-funded primary school typically has 1–3 classes, depending on the size and location of the school, with class sizes typically between 20 and 30 (mean of 27, with 11% of classes having over 30 pupils [27]). The school year begins in September and consists of six terms, each approximately 6–7 weeks long and separated by school holidays, with the longest being 6–7 weeks over the summer. We consider an intended population of a single year group, a main outcome of accelerometer-measured average weekday minutes of moderate to vigorous physical activity (MVPA), and a study duration of one school year (September-July), with baseline measures collected at the end of the previous school year in Terms 5–6 (May-July).

### Overview of simulation approach

Two of the studies presented in this paper rely on simulation methods to explore statistical properties of the design, including power. For complex designs such as this, standard sample size formulae do not always allow the combination of practical constraints that are needed. A simulation approach offers flexibility for non-standard designs [28, 29], for example to explore the impact of seasonality or different assumptions about pupil recruitment over time, and can be used both to compare different choices of design (Study 2), and to calculate power for the final design (Study 3). The general approach is as follows [28]:

1) **Data generating model**: This is a statistical model describing the relationship between intervention status and outcomes which specifies the different sources of variability. The intervention effect and other parameters are all assumed to be known.

2) **Generate a random simulation of data**: A dataset is generated from the data generating model, with outcomes (weekday MVPA) for each pupil in each school at each time point. It depends on the specific stepped wedge configuration and each simulation will be different due to the variability described in the data generating model.

3) **Fit the analysis model**: We fit a statistical model to the simulated data, estimate the intervention effect and test whether to reject the null hypothesis. As these are simulated data, we know that there is a true non-zero effect, but the estimated effect size will differ due to variability between datasets, and in some cases will be sufficiently small that the null hypothesis is not rejected, even though the alternative hypothesis is true (Type II error).

4) **Repeat the simulation process a large number of times**: The proportion of simulated datasets for which the effect size is statistically significant provides a simulation-based estimate of the power. Other summaries, such as the bias or variability in the effect estimate. can also be calculated. The larger the number of simulations, the more precise these estimates are. For example, 10,000 simulations will be sufficient to estimate power to within two decimal places.

We used mixed effect models [15] (see Appendix A for technical details) with a continuous normally-distributed outcome for MVPA for both data generating and analysis models, which are a flexible choice for analysis of cluster-based designs [15, 19, 30, 31]. Fixed effect terms were the overall mean MVPA at baseline, the intervention effect and a term that describes trend over time. Random effect terms separated the total variation in the outcome into four components: between-school variation, school-time variation (repeated measures on the same school), within-individual variation (repeated pupils; only included for a closed or open cohort study) and residual random error [15]. While this paper does not consider class-level variation separately, additional terms can be added if necessary. For the analysis model we fitted a continuous non-linear time trend over the study duration modelled via a thin plate regression spline [32], which can incorporate both seasonality and secular trend if they exist. Note that the choice of analysis model reflects the planned analysis of the full trial. All simulations were conducted in R v4.3.2 [33] and supporting R code to calculate power for stepped wedge designs is provided on the Open Science Framework [34].

## Study 1: methods

### Participants and procedure

Study 1 was a qualitative study to identify primary school constraints and preferences that inform the choice of stepped wedge configuration for a school-based physical activity study (Fig. 2). Data were collected through 15 one-to-one semi-structured interviews with staff from 9 state primary schools in the wider Bristol area, England, between November 2023 and January 2024. Sample size was guided by qualitative information power [35], with the sample aiming to include perspectives from individuals from multiple schools with different size, governance, and socio-demographic characteristics, and occupying a range of job positions relevant to physical activity decision making and delivery within these schools. Recruitment was monitored and targeted by the research team who reflected on and discussed data and recruitment to determine whether the sample size was adequate and diverse to meet the aims of the study. School and participant characteristics are outlined in Appendix B, Table S1.

Interviews were conducted by RW, DH, and AP, and lasted between 22 and 49 min. The topic guide (see Appendix A) was developed in consultation with a state primary school teacher, and explored initial design ideas for a tailored intervention, views on the intervention tools, and views on intervention evaluation. Three interviews were conducted in person, while the remaining 12 took place via MS Teams. Interviews were audio recorded and transcribed by a University approved transcription company. The protocol for this study was published on the Open Science Framework [34]. This study was approved by the University of Bristol, Faculty of Health Science Research Ethics Committee (FREC Ref 15866 on the 19.10.2023). Informed consent was obtained for all participants who received a £25 gift voucher as recompense of their time.

### Data analysis

Conventional content analysis was utilised, where coding categories were derived from the data [36]. The analysis was supported by NVivo v13 [37]. To support reflexivity and rigour, SKS, RW and DH independently coded two transcripts. From this meeting four top-level codes were identified, including a code on the stepped wedge design, as well as several lower codes. These codes were then applied to the full dataset by SKS and additional codes were derived and applied iteratively. During the coding SKS, DH and RW met to discuss and review codes. Definitions for each were created in a codebook and where a new code was introduced all previous transcripts were reviewed. Finally, the full dataset was reviewed and coded a second time to ensure consistency and accuracy.

## Study 1: results

Analysis of the coded data generated eight key points from the primary school staff perspective that should be considered in the design of a stepped-wedge evaluation, relating to data collection and the delivery of the intervention. A summary of these, including quotes for staff, is included in Table 2.

**Table 2:** Study 1 key points and quotes

Key point		Codes	Example quotes
Data collection/stepped wedge evaluation			
1	A need to keep the administrative burden of data collection on schools low	Scheduling data collection	*“I guess the admin element would come from you guys [the research team] rather than our side. That would be it [the main issue]*,* really.”*– Participant 7: Deputy Headteacher (9.7% FSM, IMD 3)
		Practical considerations	*“Not adding additional workload to staff*,* I think*,* is the biggest one [thing the research team can do to make data collection as least disruptive as possible].”* - Participant 8: PE Lead (46.9% FSM, IMD 1)
		Practical considerations	“*It’s not like it’s*,* kind of*,* detrimental to the day*,* whether that was half an hour in an afternoon*,* it wouldn’t be a concern*.”– Participant 10: PE Lead (6.1% FSM, IMD 10)
2	Keep number of data collection time points to no more than 4–5 maximum	Scheduling data collection	*“So*,* it would be four [time points]*,* really. Potentially you could look at sticking one in May*,* but…”*– Participant 7: Deputy Headteacher (9.7% FSM, IMD 3)
		Scheduling data collection	*“I think five points over a year*,* the space of a year*,* I think is practical.”*– Participant 8: PE Lead (46.9% FSM, IMD 1)
		Scheduling data collection	“*I think that’s [either three or four data collection points throughout year 5] reasonable. Because*,* you know*,* it’s not even once a term*,* and it’s only half an hour*.”– Participant 10: PE Lead (6.1% FSM, IMD 10)
3	Space data collection time points out to once a term or every 6 weeks	Scheduling data collection	*“That’s fine*,* you could do that [data collection] termly. As long as teachers know*,* they don’t need a lot of notice for that. It’s like anything*,* the more they’re aware they won’t book a trip for that day or do something else that will suddenly cause you [the research team] problems if we say*,* ‘We can’t do that day now’.”*– Participant 3: Headteacher (9.7% FSM, IMD 3)
		Scheduling data collection	*“I think having one a term would probably be the most practical. Because you know that just one afternoon*,* or whatever it is that you need with the children*,* or with the year group*,* it can be timetabled in termly. I think if you go to eight weeks*,* it can get a little bit messy on crossovers of half-terms*,* and things like that.”*– Participant 8: PE Lead (46.9% FSM, IMD 1)
4	Avoid certain times in the school year (busy periods and assessment dates)	Scheduling data collection	*“I would suggest that December wasn’t used because it’s the month where we’re most off timetable […] other than that*,* I think any other point in the year would be fine*,* and knowing them in advance would be really useful*.” - Participant 6: Class Teacher (18.3% FSM, IMD 6)
		Scheduling data collection	*“Yeah*,* just [avoid] key assessments and probably not the first week of September or anything*,* but apart from that*,* it sounds pretty okay to manage*,* yeah.” -* Feasibility 11: Principal (35.4% FSM, IMD 4)
		Scheduling data collection	*“[In June] Year 4 have a national times table test. So*,* the first thing is avoiding that date*,* whenever that is”*– Participant 8: PE Lead (46.9% FSM, IMD 1)
		Scheduling data collection	*“Yeah*,* definitely*,* you have to avoid SATs week. […] And key assessment weeks. I mean I’d find that quite stressful if it was like suddenly we’ve got to do this in this week in May*,* when actually all of the staff are actually helping out with that assessment. So I think we need to be able to reassure schools that it won’t be these weeks.”*– Participant 11: Principal (35.4% FSM, IMD 4)
		Scheduling data collection	*“The only thing*,* I think*,* especially with us being a small school*,* is around SATs week*,* avoiding the lead-up to that. In bigger schools*,* it wouldn’t make such a difference*,* because their Year 5 and Year 6 are separate*,* but obviously*,* for us*,* they’re in one class.”*– Participant 15: PE Lead (7.1% FSM, IMD 7)
**Intervention delivery**			
5	Lack of flexibility in when the intervention begins in the school year is acceptable	Scheduling implementation	*“I think teachers and schools are so set on dates and stuff that we’re quite rigid for things too*,* so I think that [inflexibility on when the intervention would begin] would be fine.”*– Participant 6: Class Teacher (18.3% FSM, IMD 6)
6	Minimum notice of a term/6 weeks for the start of the intervention to resolve timetabling issues	Perceptions of feasibility	*“Schools are incredibly routine*,* very regimented. The more notice we have with stuff*,* the better. Saying that*,* we’re very good at turning things around quickly. If I can put it in diaries that we know you’re coming in this week or even to the day*,* that will help. […] I think we’d realistically needs a term’s notice*,* which is six weeks*,* as a minimum for us. Most terms are six to eight weeks. Obviously*,* now we’re at the end of term two. If you asked me to put something in term three*,* I’d really struggle. I might be able to do it*,* but it might be a struggle.”*– Participant 3: Headteacher (9.7% FSM, IMD 3)
7	Lead in time required varies by intervention component	Scheduling implementation	*“A term [notice] would be okay*,* but much shorter than that is quite short.”*– Participant 15: PE Lead (7.1% FSM, IMD 7)
		Scheduling implementation	*“So I’d say*,* yeah*,* probably at least a term’s preparation for anything that involves some training. Anything that’s bigger than that*,* a school process or changing when this happens or that*,* would probably be a yearly thing. […] If it was a very small tweak to a PE lesson*,* or something like that*,* or putting some new equipment out at playtime then that could be done almost straightaway. Yeah*,* things do happen and can happen quite quickly as well.”*– Participant 9: Class Teacher (7.1% FSM, IMD 7)
		Scheduling implementation	*“[Organising and setting up external club provision takes] not long at all. We’ve got a very effective admin team. The issue might be if you need a hall*,* a playground or something to do something physical*,* it’s just scheduling it. In real terms we could easily get something like [external provision] set up probably within two weeks. It wouldn’t take long at all because you’ve got your captive audience. We want to do something in two weeks’ time*,* it would be timetabling issues and what do we swap around*,* what do we need to move? A couple of weeks to make that happen*,* realistically. Longer is always good*,* but you could do it as quickly as that.”*– Participant 3: Headteacher (9.7% FSM, IMD 3)
8	Concern for the impact of season on when the intervention begins	Seasonality	*“Even when we have a wet play*,* for example*,* that would be another maybe factor that may bring your levels or your readings down on those because a wet play day*,* you’re just stuck in the classroom all day*,* and we’ve had three or four of them this year.”*– Participant 12: Class Teacher (60% FSM, IMD 1)

FSM (Free School Meals) - UK government scheme to provide free meals at school to children from low-income families, % of pupils in receipt of these;

IMD (Index of Multiple Deprivation) - a measure of area deprivation based on school postcode, with 10 being least deprived and 1 being most deprived

### Data collection/Stepped wedge evaluation

In general, keeping the administrative burden on schools to a minimum was important for primary school staff. They found the proposed data collection procedures (recruiting pupils and organising the accelerometer distribution and collection) to be low burden to the school (Table 2, point 1), and so the proposals for data collection at multiple time points was considered feasible and acceptable. Across school staff it was expressed that data collection at three timepoints within a year would be acceptable with four as a maximum, but that any more would become burdensome for schools Table 2, point 2). School staff expressed that they would prefer to know specific days for data collection as far in advance as possible, whilst some schools mentioned that they would prefer a term’s notice or 6 weeks as the minimum, in order to resolve any timetabling issues and avoid any cross-over with the half-term holidays (Table 2, point 3). Furthermore, school staff suggested avoiding data collection at certain times in the school year: the end of terms, particularly in December, which are often off timetable; September, as a period of change, and assessment times e.g. June for the Year 4 National Multiplication Assessment and the Year 6 Standardised Assessment Tests (Table 2, point 4).

### Intervention delivery

School staff understood that due to the parameters of the stepped wedge design they would have limited notice and no flexibility as to when their school would receive their intervention. However, staff found this acceptable as they are accustomed to rigid timetabling (Table 2, point 5). School staff expressed a preference for a term lead-in as a minimum to implement the new interventions, depending on the specific components, as anything less could put strain on the school (Table 2, point 6). Some timetabling, such as plans for staff training on INSET days (days where schools are closed to pupils for staff training) are scheduled up to a year in advance., although this varied across schools. Some intervention components, such as extracurricular activities, are more flexible, as they are planned on a term-by-term basis, whilst organising external provision can be introduced within a two-week time period (Table 2, point 7). Finally, staff expressed a concern of the impact of seasonality on delivering the intervention and in turn the data collected. For example, heavy rain often leads to children remaining inside for ‘wet play’ days with some facilities and spaces such as fields out of bounds for a time (Table 2, point 8).

## Study 2: methods

Study 2 was a simulation study to choose an appropriate stepped wedge configuration that met the school constraints identified in Study 1 (Fig. 2). This specific configuration will be used to explore power under different scenarios in Study 3 for final sample size estimates.

### Practical considerations

Table 3 shows the key school considerations from Study 1, along with other statistical and practical considerations, and their implications for the study design. We balanced the different criteria based on priority. Specifically, we chose a measurement period (that is, the time between each step) of one school term, with an implementation period of one term, which required us to consider incomplete designs with five steps (the maximum number of steps possible). We then restricted to a total of three measurements across the year, plus one at baseline, a data collection burden reported as acceptable to schools. This resulted in five sequences, with interventions implemented during Terms 1–5. We further required that intervention measurements immediately followed the implementation period (designs which tend to have higher power [38]), and that there were no more than two measurements in consecutive terms (lower burden to schools). This resulted in 256 possible configurations (see Appendix A for details).

**Table 3:** Key practical considerations and implications for study design

Consideration	Implications for study design	Priority
**School considerations (from Study 1)**		
Must work around school terms	Minimum measurement period is one school term	1
No more than one measurement per term		
Intervention to start at beginning of a term	Steps occur at beginning of term - a maximum number of 6 steps per school year	1
One term needed to implement	Implementation period required of 1 term– requires incomplete design, and reduces maximum number of steps by one	1
Maximum of 4–5 measurement points per school year (3–4 preferable)	Restricts total number of measurements per school per year– may require incomplete design	1
School disruptions at certain times of year (e.g. end of term, Christmas, exam period)	Ideally avoid designs with more schools during busy times	2
**Statistical and Logistical considerations**		
Seasonality in physical activity outcomes	Need to ensure time is modelled correctly, and consider the impact of time on the design	1
Baseline data collection is more intensive	Spread baseline data collection over two terms Keep data collection spread over time periods	2
Difficulties in managing large volumes of data collection at any time		
Class sizes give an upper limit on pupil numbers per school	Allow pupils to join and drop out at different times to maximise participant numbers - open cohort design	2

Priority: 1 = essential, 2 = desirable

### Simulations

We simulated power for each of the 256 configurations, keeping the school and pupil sample sizes, outcome, intervention effect and correlation estimates fixed as follows:

• A true intervention effect of 5 min increase in MVPA (similar to that observed in previous children’s physical activity interventions [39]), with standard deviation of 23 min [40].

• A closed cohort of 27 pupils per school (mean class size for English primary schools).

• Correlations for weekday MVPA in primary schools of school ICC = 0.08, CAC = 0.75, IAC = 0.45 [40].

Standard formulae [15] estimate that 15 schools would be required to achieve 85% power in the complete five-step design using these parameters; we therefore used 15 schools in these simulations, with a balanced allocation of three schools to each sequence. These power estimates provide relative rather than absolute comparisons, assuming that changes in the input parameters are approximately independent of each other (consistent with previous formulae [15, 21, 29, 41]). Note that Study 3 will explore how these parameters affect power directly for the chosen configuration.

We generated 10,000 simulations for each configuration. For each configuration we then calculated the average intervention effect, percentage bias (estimate minus true value, as a percentage of the true value), the average standard error of the estimate and the power of the design (proportion of simulations that reject the null hypothesis of no effect). In an incomplete design, the measurement and non-measurement periods for each sequence are time-specific, and so may be impacted by seasonal trends in the outcome. To explore this, we ran simulations firstly with no time effect, and then again using a seasonal trend that varied by 4 min average MVPA between winter and summer (Appendix B, Figure S1) using a cyclic cubic spline [42] based on a previous study in England [43]. We plotted the average standard error against bias for each specification and compared with and without time trends.

To select a suitable configuration, we balanced statistical properties with the qualitative findings from Study 1 to ensure the design would be acceptable to schools. We first identified those designs which had low variability and low bias (and hence high power) when there was no underlying seasonality, and then selected from those a shortlist of the ten designs with lowest bias when seasonality was present. These were individually assessed with respect to the considerations in Table 3, by considering the total number of schools needing data collection per term from a logistical perspective, the number of schools needing data collection at pressured times of the year (e.g. assessments in June), and the number of consecutive measurements to minimise the number of times schools would have measurements in two consecutive terms. We selected one design to take forward to Study 3.

## Study 2: results

### Comparison of design configurations

Power for the complete five-step design was 85%. Without seasonality, power was lower for incomplete designs, with large variations between 62 and 80% for the 256 incomplete designs considered. The simulations indicated that terms 2–4, where there were schools under both control and intervention conditions, were most important in determining the power, with higher power when there was a mix of control and intervention measurements. We identified a group of 48 designs with high power 76–80% (Appendix B, Figure S2).

**Fig. 3:**
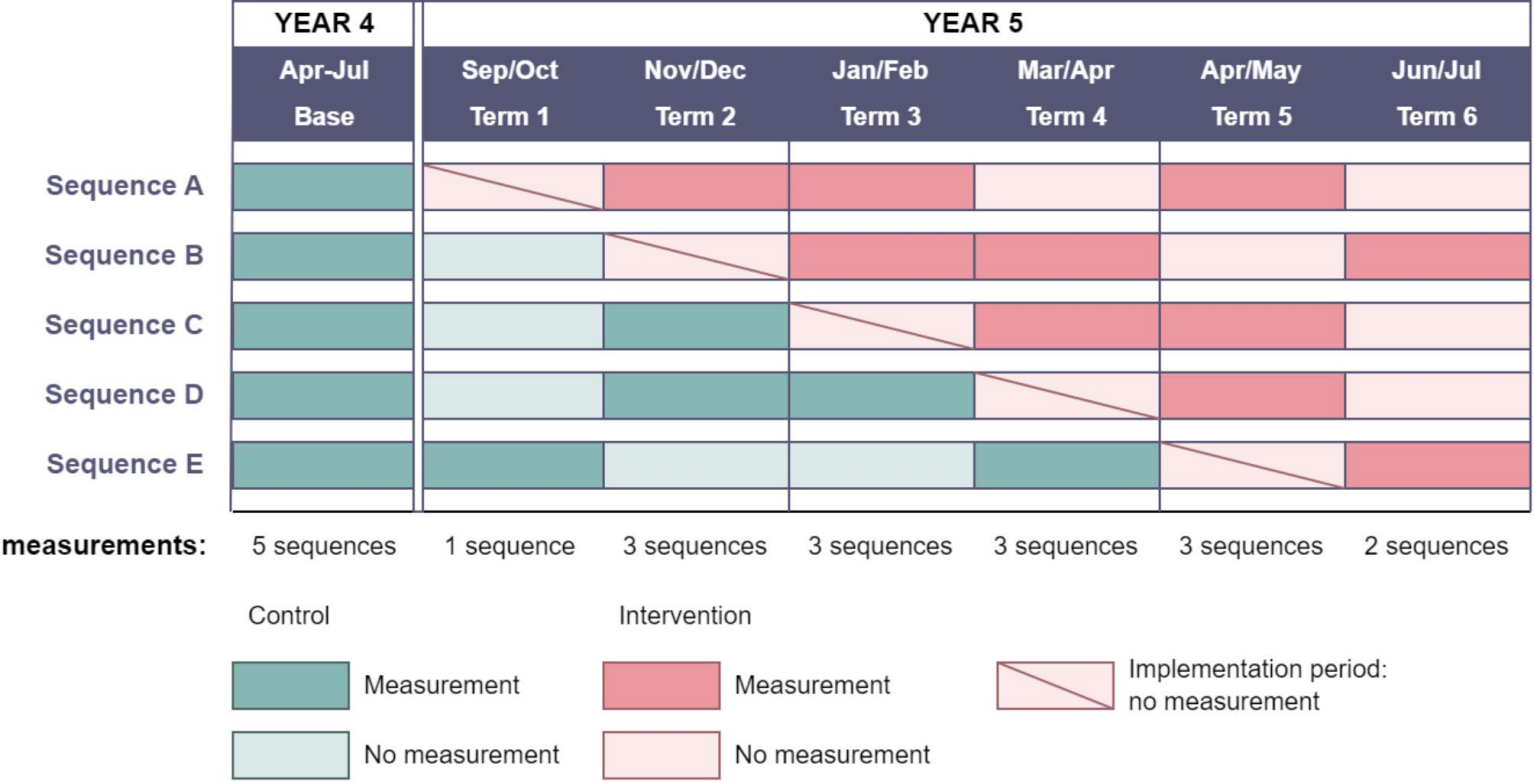
Final choice of stepped wedge design

Seasonality in the outcome introduced bias of between − 10% and + 7% (Appendix B, Figure S3) with the intervention effect both under and over-estimated. The 48 high-power designs identified above also performed well when the seasonality term was added, with bias typically under 5%, and smaller standard errors than other designs. Designs with the maximum number of measurements and a mix of control and intervention conditions in terms 2–4 had the lowest bias. In addition, bias was lower for designs with a spread of measurements under either condition across the whole study duration. We identified ten of the best designs and chose the final design (Fig. 3) by considering school and other practical issues identified through Study 1. See also Appendix B, Figure S4 for some examples of other configurations.

## Study 3: methods

Study 3 (Fig. 2) was a simulation study to explore the statistical power for the specific stepped wedge design configuration design chosen in Study 2 (Fig. 3). We simulated data for different scenarios designed to (a) explore the impact of an open cohort on the power; (b) to reflect uncertainty in the school correlation parameters (ICC and CAC); and (c) to produce different combinations of plausible intervention effects and numbers of schools to determine the number of schools required to detect a specific intervention effect.

To explore the impact of an open cohort, we assumed a ‘closed population’ scheme [21], that is, repeated sampling from a fixed population of eligible pupils. We considered eligible populations of size 30 and 60 (corresponding approximately to one and two classes per year group). The open cohort scheme was characterised by an overall study recruitment rate (percentage of eligible pupils participating at any time), and the retention rate (percentage of pupils participating at one measurement period who also participate in a second measurement period). The latter is sometimes characterised in terms of the attrition or ‘churn’ rate [44], where churn = 1 - retention. This scheme will result in relatively stable cluster sizes across time. We further fixed the recruitment and retention rates to be equal, and chose values to result in a range of participating pupils at each point between 33% and 83% as follows:

• Recruitment/retention rate = 58%: 33% of eligible pupils at each time point (10/30 or 20/60).

• Recruitment/retention rate = 71%: 50% of eligible pupils at each time point (15/30 or 30/60).

• Recruitment/retention rate = 82%: 67% of eligible pupils at each time point (20/30 or 40/60).

• Recruitment/retention rate = 91%: 83% of eligible pupils at each time point (25/30 or 50/60).

Values for the correlations (ICC, CAC and IAC) were based on previous estimates [40], with values of 0.08 and 0.10 for the ICC corresponding to the point estimate and upper 95% confidence interval bound, and values of 0.75 for the CAC and 0.45 for the IAC. We explored true intervention effects between 5 and 10 min increase in MVPA, with a standard deviation of 23 min [40], and included a background seasonal trend as described in Study 2 (see (Appendix B, Figure S1). We generated 50,000 simulations per scenario (to allow for additional Monte Carlo error in sampling the open cohort) and estimated power for 15 and 20 schools (with equal number of schools allocated to each sequence). Finally, for comparison we calculated the power for an equivalent CRCT of follow-up outcome adjusted for baseline, with baseline-adjusted ICC values of 0.06 and 0.08 [40].

## Study 3: results

**Fig. 4:**
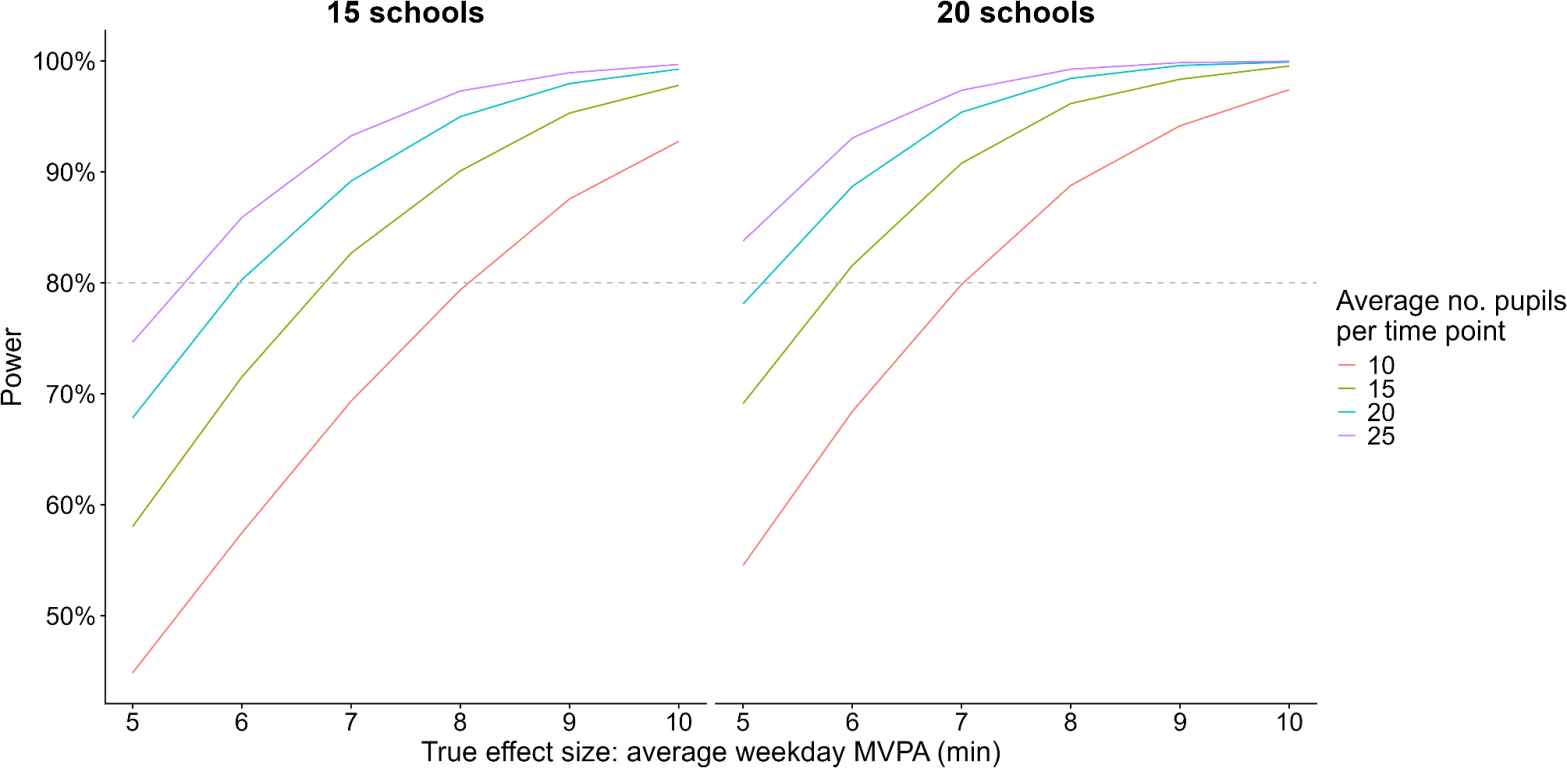
Power for final stepped wedge design for different effect sizes and sample sizes (assuming a year group size of 30)

We calculated the power for intervention effects of 5–10 min difference in weekday MVPA, under a range of scenarios for the chosen configuration (Fig. 4 & Appendix B, Table S2). Power was higher for more schools and when there were more pupils measured at each measurement point, with the latter depending on both the overall recruitment rate and the retention rate. For 15 schools, this design would have power of 80% or higher to detect a 7 min difference in MVPA for an eligible year group size of 30 pupils per school and at least 50% of pupils per school at each measurement period, or a 6 min difference for year group sizes of 60 eligible pupils. For 20 schools, we would be able to detect a 6 min difference for a 30pupil year group per school, or 5 min for a 60-pupil year group, provided there were at least an average of 50% of eligible pupils measured per school at each measurement period. For comparison, we also calculated the power for a comparable CRCT, with analysis of the outcome adjusted for baseline (Appendix B, Tables S3 & S4). Power was substantially lower, with the comparable CRCTs able to detect a minimum difference of MVPA of 9–10 min/day. A CRCT designed to detect 6 min difference in MVPA with 20 pupils at each time would require 42 schools (84 measurements in total), compared to 15–16 schools (60–64 measurements in total) for the final stepped wedge design.

## Discussion

This paper has explored practical considerations which need to be considered in designing a stepped wedge trial of school-based physical activity interventions, and how those considerations affect the choice of stepped wedge configuration and the resulting power. While simpler stepped wedge designs may be difficult to implement in practice, we found that considering more flexible alternatives such as incomplete designs made stepped wedge trials more feasible. While there were losses in statistical power between complete and incomplete designs, with careful choice in the configuration to ensure a mix of control and intervention measurements at any given time, these losses were minimised. For example, the final configuration chosen here had power only slightly lower than the equivalent complete design (92% compared to 95%). This slight reduction in power was offset by a number of benefits: substantially fewer schools were required than in the similarly powered CRCT, it involved approximately 75% fewer measurements, and these were spaced out over the study duration. The latter is appealing to schools as well as being logistically easier, especially in the presence of seasonality when CRCT measurements in all schools need to be taken close in time. The estimate of 42 schools required for a similarly-powered CRCT is based on a robust ICC estimate from multiple pooled datasets [40], which is higher than typically used for power calculations. As a result, many published studies use fewer schools, but may be underpowered, especially as CRCTs are more sensitive to mis-specification of the ICC than stepped wedge designs. This all suggests that if done well, the stepped wedge trial has the potential to be a viable design for evaluating physical activity interventions in schools, although it still needs to be tested in practice. For the specific example considered here, detecting a minimum difference of 5–6 min in weekday MVPA (comparable with children’s physical activity interventions [39]), should be achievable under a range of realistic scenarios.

Table 4 lists the key considerations for designing a stepped wedge trial in practice. Qualitative data showed that schools were generally supportive of the design and willing to support recruitment and repeated data collection, but researchers accommodating school structures and timetabling was perceived as essential. The complexity of the stepped wedge design means that these issues need to be considered earlier in the process than for CRCTs, before the design is finalised. While this paper has focused on schools, the same applies to other settings; the specific features and constraints of the setting need to be understood and built in at the design stage. Incomplete (and similar designs such as hybrid [45], staircase [46], or dog-leg designs [47]), offer the flexibility to adapt to practical settings, and the variation observed in power between the different configurations suggests that within those constraints, careful choice of design can improve statistical power still further. For example, the spacing of measurements in incomplete designs was key to identifying more powerful designs and it was important to have a mixture of both control and intervention measurements across multiple measurement periods. This results in a concentration of measurements around the intervention implementation, balanced with ‘hot-spots’ in the corners of the design [48].

**Table 4:** Key considerations for designing a stepped wedge trial

Consideration	Why this is important
Total number of measurements acceptable	Requires either a design with fewer steps, or an incomplete design • Both reduce power (especially designs with fewer steps) • Seasonality increases variability Incomplete designs: • can be more complicated e.g. calculating power Seasonality can introduce bias so important to consider the timing of measurements
Duration of measurement period	• Very short measurement periods can be logistically difficult to ensure all data collection occurs within the required timeframe • Larger measurement periods (for the same study duration) will reduce the maximum number of steps possible, which reduces power Very wide measurement periods will introduce more variability and possible bias when seasonality is present
Implementation period	Requires an incomplete design: • can be more complicated e.g. calculating power • Seasonality can introduce bias so important to consider the timing of measurements May also affect the duration of measurement period (see above) as the implementation time will need be one or more measurement periods
Other restrictions on timings	• Constraints mean that the most powerful configurations may not be feasible • When seasonality is present, may introduce variability and/or bias due to gaps at key times which affect coverage and control/intervention balance (see below)
Coverage of full study duration	For incomplete designs, measurements should cover the full study duration (i.e. avoid staggered designs) to accurately estimate any time trends or seasonality
Control-intervention balance within measurement periods	Where possible, incomplete designs should have a balance of measurements under both control and intervention conditions to be able to separate time trends/seasonality from intervention effects
Modelling of time	• All stepped wedge analyses should include a time effect, but how this is modelled becomes more important in the presence of time trends • Seasonality and/or time trends increase variability, which will reduce power– better modelling of time will reduce this extra variability Seasonality and time trends can introduce bias if not modelled correctly which can be substantial e.g. larger than effect sizes
Class sizes	• restricts the maximum number of pupils possible to recruit, which reduces maximum power than can be achieved • smaller class sizes may therefore require more schools, and/or more pupils with repeated measures and so higher retention rates are needed (see below)
Repeat measurements: Cross-sectional, open or closed cohort	• repeated pupil measures (open or closed cohort) will increase power • may be harder to retain existing pupils than recruit new ones, especially over longer study durations closed cohort may not be possible in some situations e.g. longer studies when pupils move up a year/to a different school
Recruitment and retention rates	• dictates the number of pupils per school in each measurement period • more pupils increases power

This paper has focused on the design for an evaluation of an intervention aimed at a single year group, where the eligible population within a school is small and constrained by class sizes. Evaluations of multiple year groups will have larger total eligible populations, but the between-class variation will also be larger and ideally should be included in the model, and so within-class sample sizes will similarly be an issue, especially to ensure representativeness across the age range. Therefore, we propose that recruitment strategies should focus on an open cohort design to get the benefit of both overall participant numbers and repeated measurements, a balance between cross sectional and closed cohort criteria [21]. The simulations in this paper suggest that when the eligible population is relatively small (30–60), power is primarily driven by overall numbers participating at each measurement point, with 20–25 pupils per school giving similar power for year group sizes of both 30 and 60, even though fewer will be repeated measurements in the latter. Increasing either the recruitment rate or the retention rate will increase power, and thus the higher the overall recruitment rate, the lower the retention rate needed for the same power. Thus increased overall participation can potentially mitigate lower power if retention rates decline over time, as well as maintaining overall representativeness. This suggests that strategies should initially focus on increasing the overall study recruitment rate to 60–70% and then focus on retaining pupils.

We found that seasonal trends in the outcome measure had substantial impact on bias in the intervention effect estimate for the incomplete designs considered here, which highlights the importance of modelling seasonal patterns appropriately when they are present. In particular, we saw both under and over-estimation, between − 13% and + 8%; in the latter case, power will be artificially high, and so both bias and precision need to be considered when simulating designs which include time effects. Time is typically treated as a nuisance factor in stepped wedge designs, with a greater focus on estimating how intervention effects change over time [17, 49], rather than on background trends. A previous simulation study [18] that focused on secular rather than seasonal patterns, recommended using discrete time effects when there is no a priori information about time trends. However, in this case, children’s physical activity is known to be seasonal in many countries, including the UK [26, 50], and so we modelled a continuous nonlinear time effect. Configurations that performed well, with both low bias (< 5%) and high precision, were those that were able to separate seasonality from intervention effects, and so had good overall coverage across the study duration (ability to estimate the overall trend) plus a good mix of intervention and control measurements in each measurement period (ability to estimate the intervention effect). While this suggests that seasonality may be less of a concern if the anticipated intervention effect is orders of magnitude higher than the seasonal variation, further work in this area is needed. Guidance on the design and analysis of stepped wedge trials when seasonal and/or secular trends are present would support the use of this study design in a wider range of settings.

While the final design presented in Fig. 3 is specific to our planned study, the process and many of our findings are generalisable to a wide range of contexts, including other primary and secondary school settings outside the UK. Table 4 gives some guidance on the importance of different issues under different circumstances. For example, a common theme throughout this paper is the impact of seasonal trends, which is particularly important when the difference in children’s physical activity between the highest and lowest points is of a similar order of magnitude to meaningful intervention effects (4–6 min [43]). In England, secular time trends are much smaller (approximately 2 min per year for primary school-aged children [43]), but in other situations, larger time trends will impact on the study design in a similar way to seasonality. It is always important that a time effect is included in the analysis of stepped wedge trials [51], but the specific form and considerations such as coverage and control-intervention balance will be of less importance when any time effects are less pronounced. Other factors that affect generalisability are the length of measurement and implementation periods and pupil numbers. We have used a measurement period of 6 weeks corresponding to a typical UK school term, but in other countries it may be necessary to divide longer school term structures into smaller periods to ensure a sufficient number of steps. Implementation periods will depend on the nature of the intervention, and either may not be necessary at all, potentially simplifying the design and increasing power, or be longer and thus require a longer total study duration. Finally, the importance of repeated pupil measurements and the recruitment strategies proposed above are driven by class sizes in UK primary schools, but where available pupil numbers are larger, such as in secondary schools, repeated measures will be less important and strategies should concentrate on overall recruitment rather than retention.

General patterns in power observed here, in terms of the impact of number of pupils, repeat pupils, correlations and variation in cluster size, are broadly consistent with sample size formulae that consider elements in isolation [15, 21, 29, 41], suggesting that these elements may act relatively independently of the design configuration. This suggests a hybrid strategy using existing formulae to compare and shortlist potential designs followed by simulation for fine-tuning to specific constraints. We suggest a three-step approach similar to that described in this paper. First identify any setting-specific constraints, such as implementation periods, and determine the number of steps possible. Secondly, focus on feasible configurations with good time coverage and control-intervention balance and use standard formulae [15, 21, 29] and similar tools [25, 52] to compare relative power and choose a final configuration. Finally, explore specific power/sample size for the chosen configuration, either using standard formulae if appropriate or simulation if necessary. Due to the complexity of these designs and the number of inter-dependent features, we suggest erring on the conservative side in determining the final sample size.

One strength of this paper is that it directly addresses practical rather than just statistical considerations and thus helps to bridge the gap between theory and practice. The flexibility of the simulation approach allows us to focus on very specific scenarios and combinations of circumstances that are not typically covered in the standard literature, especially where these interact either with each other or with the design (e.g. via time). However, there are a number of limitations to this approach. Firstly, it requires the use of specially written code, and running multiple simulations can be time intensive, although savings can be made by exploiting the parallelisation inherent within the code. We have also provided the code used to run these simulations to support wider use of this approach [34]. Secondly, as it requires full specification of the data generating model it is easy to inadvertently build in unacknowledged or overly specific assumptions that may limit the results. It is therefore important to explore a range of parameter values and scenarios and to treat estimates of power as a guide rather than exact, and to err on the side of caution where there is uncertainty. Finally, the results presented here are specific to a one-year study of accelerometer-measured weekday MVPA in English primary schools; other studies will need to apply the general process to their specific circumstances.

## Conclusion

Stepped wedge trials are a viable design for evaluating school-based physical activity interventions. Incomplete designs, where not all schools are measured at each point, offer the flexibility to work around practical constraints such as school terms, but it is important to involve schools early on before the design is fixed. Designs with measurements spread across the full study duration and a good mix of control and intervention measurements at each measurement period have higher power, and can reduce bias when outcomes such as physical activity exhibit seasonality.

## Electronic supplementary material

Below is the link to the electronic supplementary material.

Supplementary Material 1

Supplementary Material 2

## Data Availability

Qualitative transcripts will be made available under restricted access at the end of the research project (2027) via the University of Bristol data repository (data.bris). Study 2 and 3 use simulated data; R code to reproduce simulations is available from https://osf.io/bkm4e/.

## Abbreviations

CRCT: Clustered randomised controlled trial
ICC: Intracluster correlation coefficient
CAC: Cluster autocorrelation
IAC: Individual autocorrelation
MVPA: Moderate to vigorous physical activity
