# Supplementary material for: Designing stepped wedge trials to evaluate physical activity interventions in schools: methodological considerations
Source: Int J Behav Nutr Phys Act. 2025 Feb 25;22:22. doi: 10.1186/s12966-025-01720-z (PMC11863484; doi:10.1186/s12966-025-01720-z)
Supplement: Supplementary file 2 — Supplementary Material 2 [file 12966_2025_1720_MOESM2_ESM.docx]

Designing stepped wedge trials to evaluate physical activity interventions in schools: methodological considerations

Ruth Salway, Danielle House, Robert Walker, Simona Kent-Saisch, Alice Porter, David R Lubans, Michael Beets, Frank de Vocht and Russell Jago

Appendix B: Additional Tables and Figures

**Tables**

**Table S1**: Participant and school characteristics for Study 1

**Table S2**: Power for final stepped wedge design

**Table S3**: Power for Cluster Randomised Controlled Trial, adjusted for baseline

**Table S4**: Number of schools needed for 80% power in a Cluster Randomised Controlled Trial with outcome adjusted for baseline

**Figures**

**Figure S1:** Seasonality term assumed in the data generating model (based on data from a previous study)

**Figure S2**: Power against % bias for different incomplete designs with no seasonality, based on 10,000 simulations

**Figure S3:** Effect size standard error against bias for different incomplete designs with seasonality, based on 10,000 simulations

**Figure S4:** The remaining nine configurations considered

**Table S1: Participant and school characteristics for Study 1**

| **Participant roles** | | **n** |
| --- | --- | --- |
|  | Headteacher/Principal | 2 |
|  | Deputy Headteacher/Principal | 3 |
|  | Class Teacher | 3 |
|  | Physical Education (PE) Lead | 5 |
|  | Dedicated Physical Education (PE) Teacher | 2 |
| **School characteristics** | |  |
| *Urban/rural classifications* | |  |
|  | Urban | 4 |
|  | Suburban | 4 |
|  | Rural | 1 |
| *Free School Meal^1^ %* | |  |
|  | Below national average (23.8%) | 6 |
|  | Above national average (23.8%) | 3 |
| *School postcode Index of Multiple Deprivation^2^* | |  |
|  | 1-3 | 3 |
|  | 4-6 | 3 |
|  | 7-10 | 3 |
| *School Lower Layer Super Output Area^3^ Black Asian and Minority Ethnic^4^ %* | |  |
|  | Below national average (18.3%) | 6 |
|  | Above national average (18.3%) | 2 |
|  | No data | 1 |
| *Number of pupils in school* | |  |
|  | 0-200 | 1 |
|  | 201-400 | 1 |
|  | 401-600 | 5 |
|  | >600 | 2 |

^*^ Participants often had more than one of these roles in a school, for example, both a class teacher and the PE lead. We have categorised staff with multiple roles primarily by their PE role, then SLT role, and then class teacher.

^1^ a UK government scheme to provide free meals at school to children from low income families
^2^ a measure of area deprivation, with 10 being least deprived and 1 being most deprived
^3^ a Census-based geographical unit for England and Wales
 ^4^ defined as all ethnic groups except white ethnic group

**Table S2: Power for final stepped wedge design (based on 50,000 simulations)**

|  |  | ICC=0.08^1^ | | | | | | ICC=0.10^1^ | | | | | |
| --- | --- | --- | --- | --- | --- | --- | --- | --- | --- | --- | --- | --- | --- |
|  | MVPA effect size: | 5 min | 6 min | 7 min | 8 min | 9 min | 10 min | 5 min | 6 min | 7 min | 8 min | 9 min | 10 min |
| 15 schools | | | | | | | | | | | | | |
| Average pupils per school per measurement period | | | | | | | | | | | | | |
| 30 eligible pupils | | | | | | | | | | | | | |
|  | 10 (33% of eligible) | 45% | 57% | 69% | 79% | 88% | 93% | 45% | 57% | 69% | 78% | 86% | 92% |
|  | 15 (50% of eligible) | 58% | 72% | 83% | 90% | 95% | 98% | 58% | 70% | 81% | 89% | 95% | 97% |
|  | 20 (67% of eligible) | 68% | 80% | 89% | 95% | 98% | 99% | 66% | 79% | 88% | 94% | 97% | 99% |
|  | 25 (83% of eligible) | 75% | 86% | 93% | 97% | 99% | >99% | 73% | 84% | 92% | 96% | 99% | 99% |
| 60 eligible pupils | |  |  |  |  |  |  |  |  |  |  |  |  |
|  | 20 (33% of eligible) | 65% | 78% | 88% | 94% | 98% | 99% | 65% | 77% | 87% | 93% | 97% | 99% |
|  | 30 (50% of eligible) | 78% | 89% | 95% | 98% | 99% | >99% | 77% | 87% | 94% | 97% | 99% | >99% |
|  | 40 (67% of eligible) | 84% | 92% | 97% | 99% | >99% | >99% | 82% | 91% | 96% | 98% | 99% | >99% |
|  | 50 (83% of eligible) | 87% | 95% | 98% | 99% | >99% | >99% | 85% | 93% | 97% | 99% | >99% | >99% |
| 20 schools | | | | | | | | | | | | | |
| Average pupils per school per measurement period | | | | | | | | | | | | | |
| 30 eligible pupils | | | | | | | | | | | | | |
|  | 10 (33% of eligible) | 55% | 68% | 80% | 89% | 94% | 97% | 54% | 68% | 79% | 88% | 93% | 97% |
|  | 15 (50% of eligible) | 69% | 82% | 91% | 96% | 98% | >99% | 68% | 81% | 90% | 95% | 98% | 99% |
|  | 20 (67% of eligible) | 78% | 89% | 95% | 98% | >99% | >99% | 76% | 87% | 94% | 98% | 99% | >99% |
|  | 25 (83% of eligible) | 84% | 93% | 97% | 99% | >99% | >99% | 82% | 92% | 97% | 99% | >99% | >99% |
| 60 eligible pupils | |  |  |  |  |  |  |  |  |  |  |  |  |
|  | 20 (33% of eligible) | 76% | 87% | 94% | 98% | 99% | >99% | 74% | 86% | 93% | 97% | 99% | >99% |
|  | 30 (50% of eligible) | 87% | 97% | 98% | >99% | >99% | >99% | 85% | 93% | 97% | 99% | >99% | >99% |
|  | 40 (67% of eligible) | 91% | 97% | 99% | >99% | >99% | >99% | 89% | 96% | 98% | >99% | >99% | >99% |
|  | 50 (83% of eligible) | 94% | 98% | 99% | >99% | >99% | >99% | 91% | 97% | 99% | >99% | >99% | >99% |

^1^ ICC estimates (point estimate 0.08, upper 95% confidence interval bound 0.10) for weekday MVPA from:

Salway R, Jago R, de Vocht F, House D, Porter A, Walker R, Kipping R, Owen CG, Hudda MT, Northstone K *et al*: **School-level intra-cluster correlation coefficients and autocorrelations for children’s accelerometer-measured physical activity in England by age and gender**. *BMC Medical Research Methodology* 2024, **24**(1).

**Table S3: Power for Cluster Randomised Controlled Trial, adjusted for baseline**

|  |  | ICC=0.06^1^ | | | | | | ICC=0.08^1^ | | | | | |
| --- | --- | --- | --- | --- | --- | --- | --- | --- | --- | --- | --- | --- | --- |
|  | MVPA effect size: | 5 min | 6 min | 7 min | 8 min | 9 min | 10 min | 5 min | 6 min | 7 min | 8 min | 9 min | 10 min |
| 15 schools^2^ | | | | | | | | | | | | | |
| Average pupils per school per measurement period (30 eligible per school) | | | | | | | | | | | | | |
|  | 10 (33% of eligible) | 20% | 27% | 35% | 43% | 52% | 61% | 18% | 25% | 32% | 39% | 48% | 56% |
|  | 15 (50% of eligible) | 24% | 32% | 42% | 52% | 61% | 71% | 21% | 29% | 37% | 46% | 56% | 64% |
|  | 20 (67% of eligible) | 27% | 36% | 47% | 57% | 67% | 76% | 24% | 32% | 41% | 51% | 60% | 69% |
|  | 25 (83% of eligible) | 29% | 39% | 50% | 61% | 71% | 80% | 25% | 34% | 44% | 54% | 64% | 73% |
| 20 schools | | | | | | | | | | | | | |
| Average pupils per school per measurement period (30 eligible per school) | | | | | | | | | | | | | |
|  | 10 (33% of eligible) | 35% | 48% | 60% | 72% | 81% | 89% | 31% | 42% | 53% | 65% | 75% | 83% |
|  | 15 (50% of eligible) | 40% | 54% | 67% | 78% | 87% | 93% | 34% | 46% | 59% | 70% | 80% | 88% |
|  | 20 (67% of eligible) | 43% | 58% | 71% | 82% | 90% | 95% | 37% | 49% | 62% | 73% | 83% | 90% |
|  | 25 (83% of eligible) | 45% | 60% | 73% | 84% | 91% | 96% | 38% | 51% | 64% | 75% | 84% | 91% |

^1^ ICC estimates (point estimate 0.06, upper 95% confidence interval bound 0.08) for weekday MVPA from:

Salway R, Jago R, de Vocht F, House D, Porter A, Walker R, Kipping R, Owen CG, Hudda MT, Northstone K *et al*: **School-level intra-cluster correlation coefficients and autocorrelations for children’s accelerometer-measured physical activity in England by age and gender**. *BMC Medical Research Methodology* 2024, **24**(1).

^2^ power averaged for 14 and 16 schools with 1:1 allocation of control to intervention arms

**Table S4: Number of schools needed for 80% power in a Cluster Randomised Controlled Trial with outcome adjusted for baseline**

|  |  | ICC=0.06^2^ | | | | | |
| --- | --- | --- | --- | --- | --- | --- | --- |
|  | Effect size | 5 | 6 | 7 | 8 | 9 | 10 |
| Pupils^1^ | |  |  |  |  |  |  |
|  | 15 | 68 | 48 | 36 | 28 | 22 | 20 |
|  | 20 | 60 | 42 | 32 | 24 | 20 | 18 |
|  | 25 | 54 | 38 | 30 | 24 | 18 | 16 |
|  | 30 | 50 | 36 | 28 | 22 | 18 | 16 |

^1^ Average number and percentage of eligible pupils who have measurements at both baseline and follow-up

^2^ ICC estimate for weekday MVPA adjusted for baseline

ICC estimates from: Salway R, Jago R, de Vocht F, House D, Porter A, Walker R, Kipping R, Owen CG, Hudda MT, Northstone K *et al*: **School-level intra-cluster correlation coefficients and autocorrelations for children’s accelerometer-measured physical activity in England by age and gender**. *BMC Medical Research Methodology* 2024, **24**(1).

**Figure S1: Seasonality term assumed in the data generating model**

**
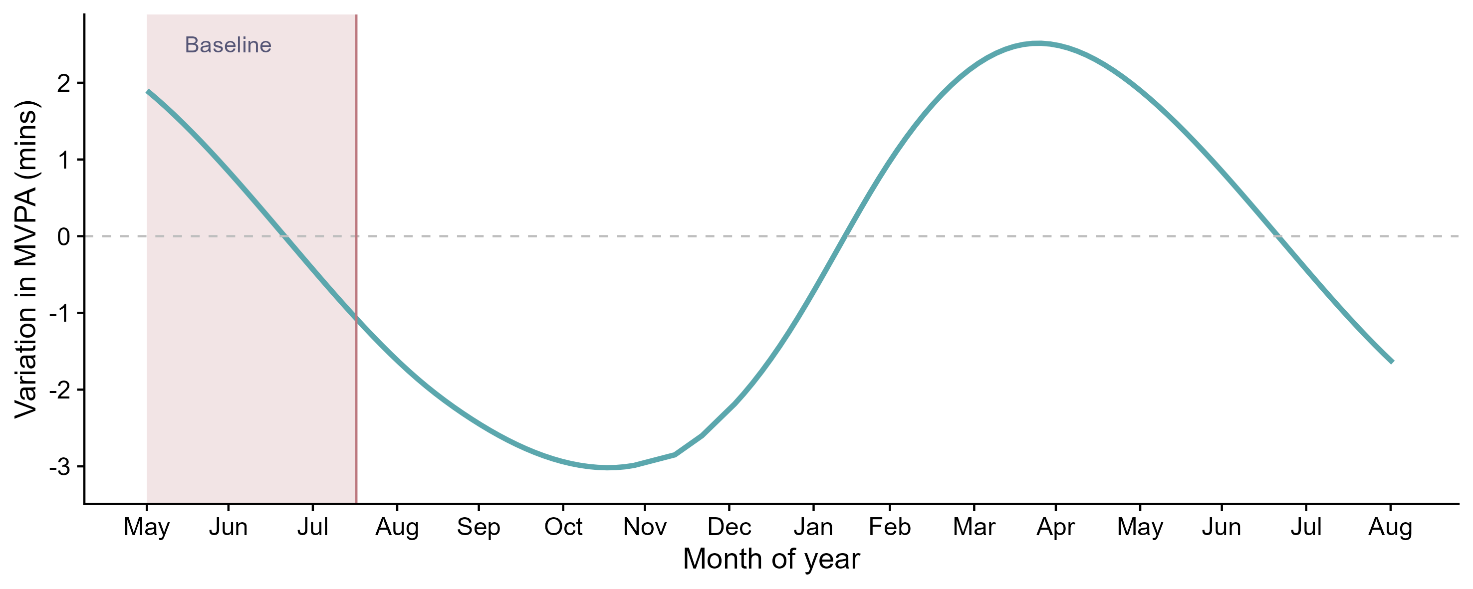
**

Seasonal trend estimated from data from:

Jago R, Salway R, Emm-Collison L, Sebire SJ, Thompson JL, Lawlor DA: **Association of BMI category with change in children’s physical activity between ages 6 and 11 years: a longitudinal study**. *Int J Obes* 2020, **44**:104-113.

**Figure S2: Power against % bias for different incomplete designs with no seasonality, based on 10,000 simulations. Dark blue points are those with highest power.**

**
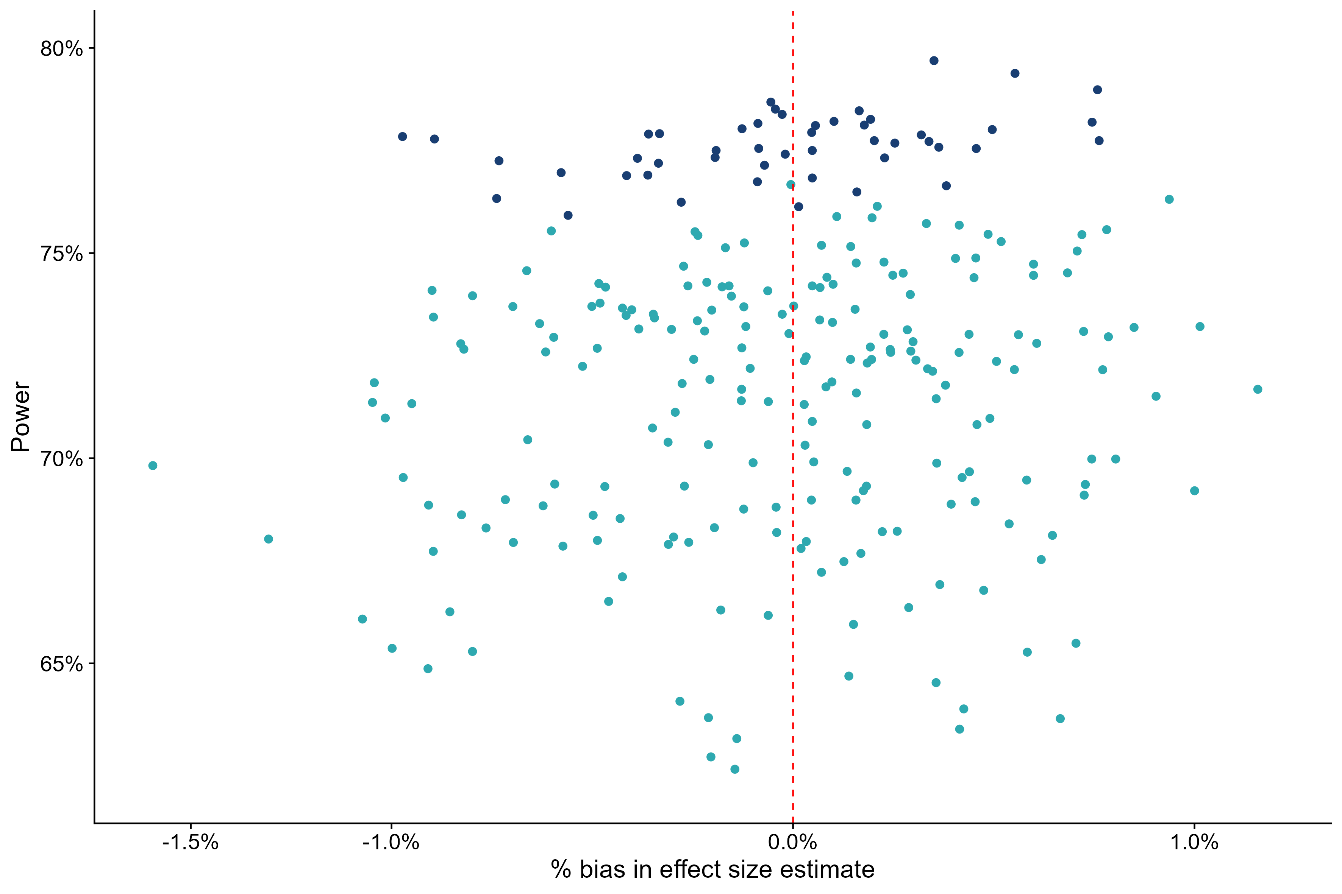
**

**Figure S3: Effect size standard error against bias for different incomplete designs with seasonality, based on 10,000 simulations. Dark blue points are those with lowest standard error and lowest bias from no seasonality model.**

**
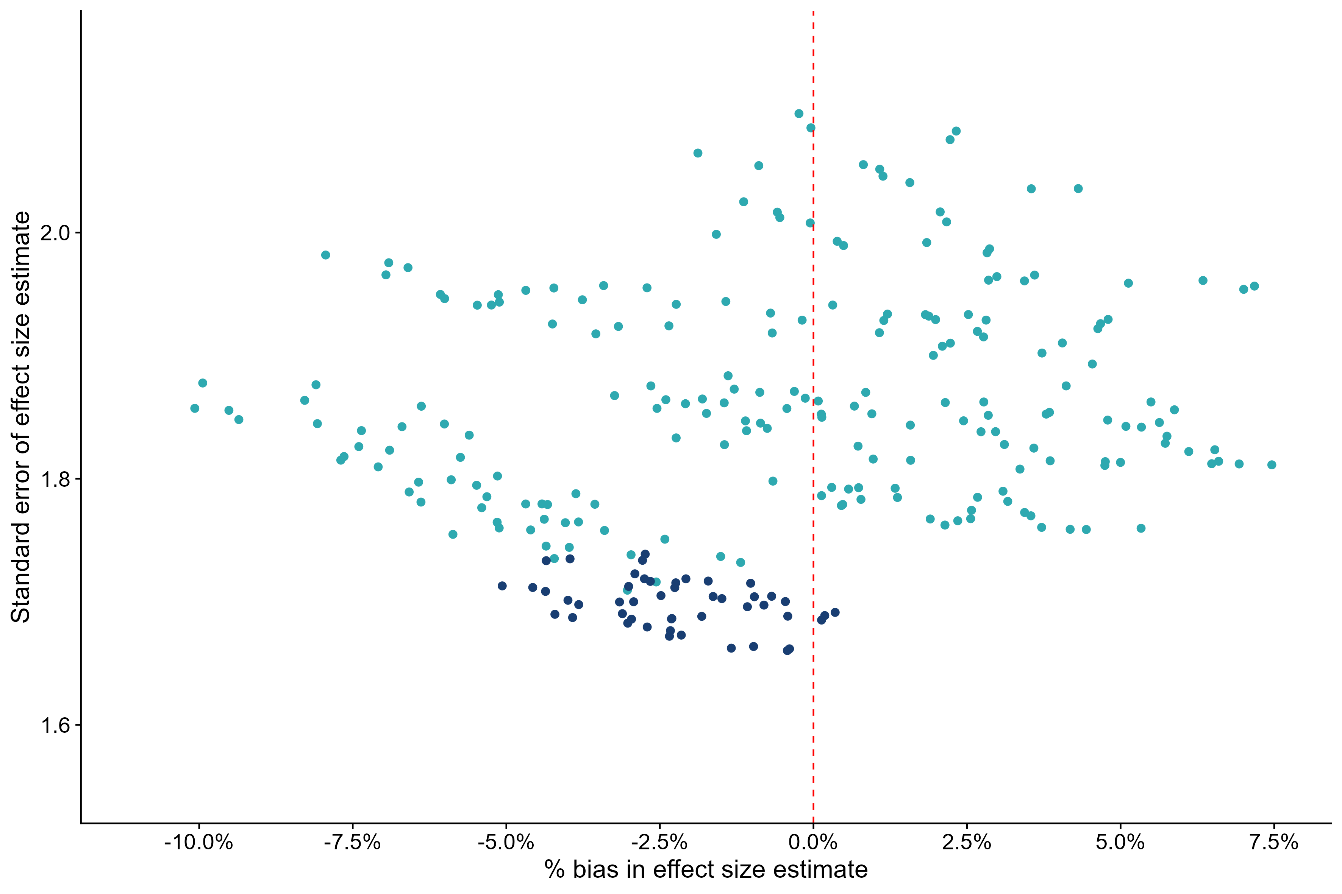
**

**Figure S4: Examples of other configurations**

**
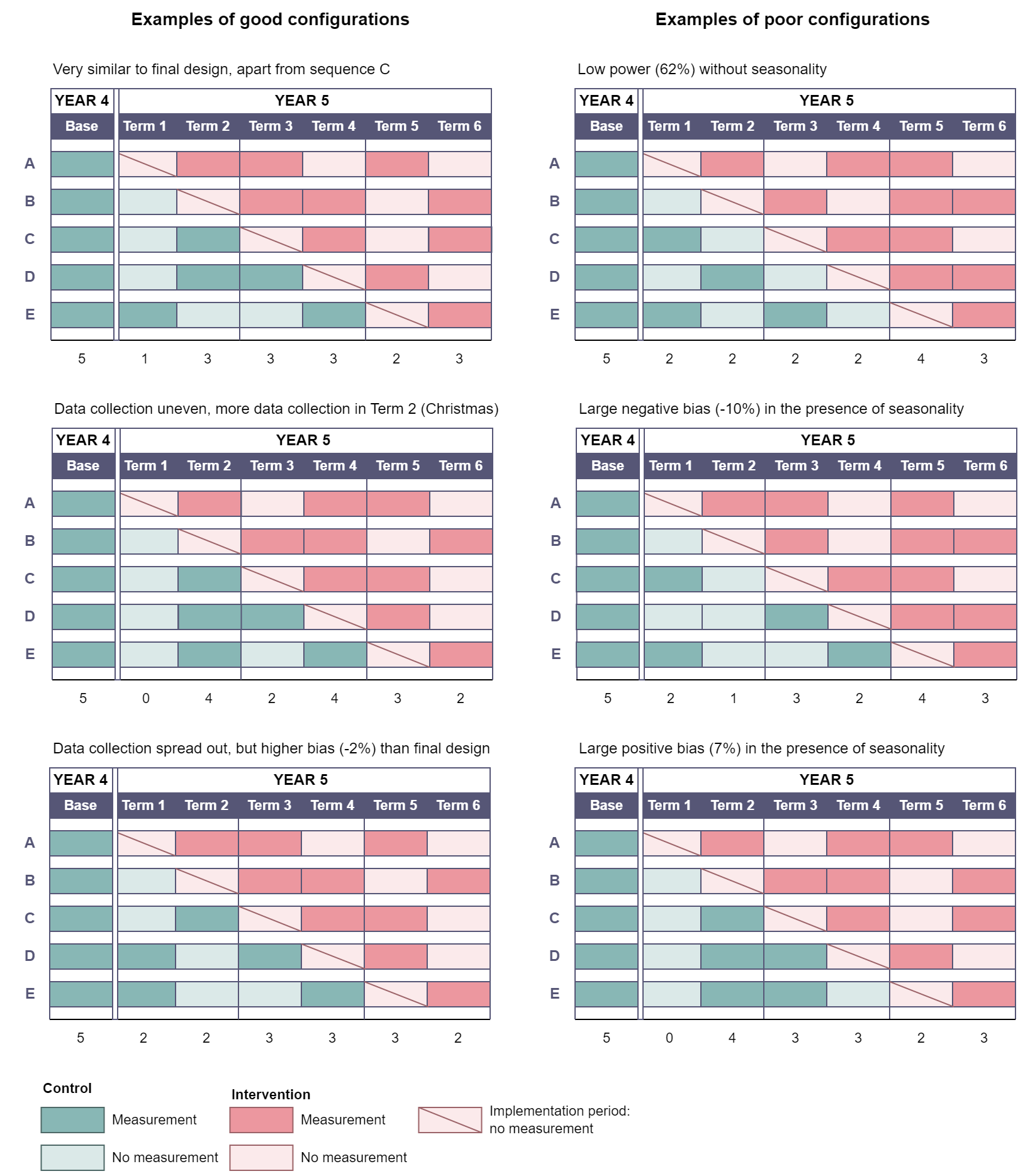
**
